# Supplementary material for: Clinical and Nutritional Effectiveness of a Nutritional Protocol with Oligomeric Enteral Nutrition in Patients with Oncology Treatment-Related Diarrhea
Source: Nutrients. 2020 May 25;12(5):1534. doi: 10.3390/nu12051534 (PMC7284999; doi:10.3390/nu12051534)
Supplement: Supplementary file 1 [file nutrients-12-01534-s001.pdf]

Supplementary

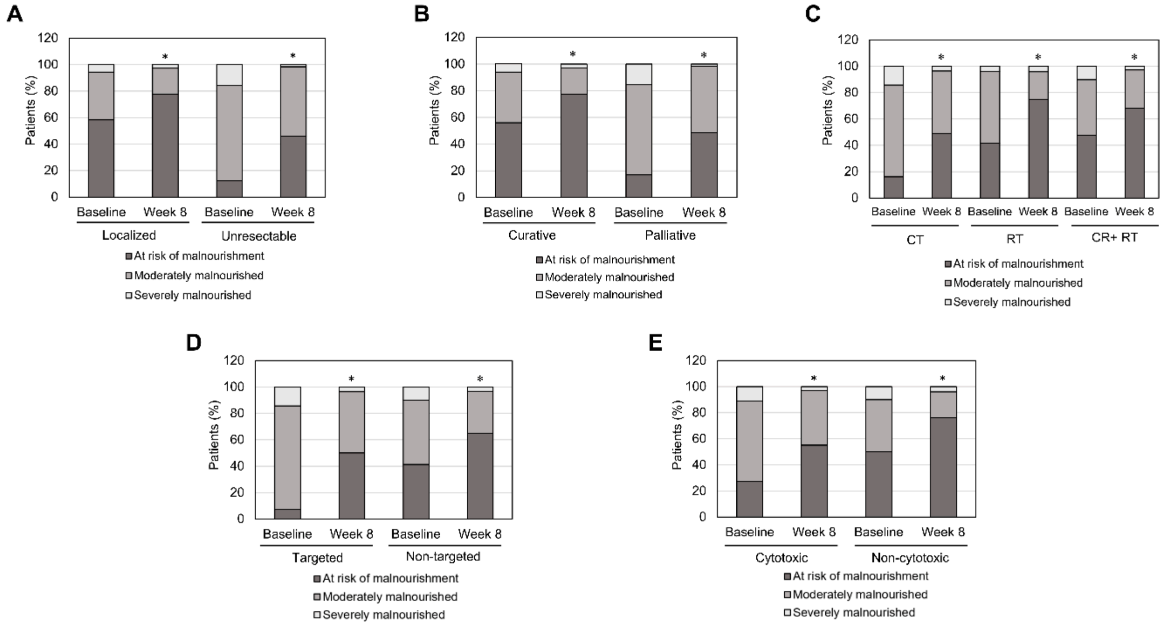

Figure S1. Change in nutritional status after 8 weeks of oligomeric enteral nutrition (OEN) support by patient subgroups. The stacked bars show the proportion of patients at risk of malnourishment and with moderate or severe malnourishment at baseline and after 8 weeks following the OEN protocol by patient subgroups: (**A**) localized vs. unresectable; (**B**) curative vs. palliative; (**C**) chemotherapy, radiotherapy, or both; (**D**) targeted vs. non-targeted; and (**E**) cytotoxic vs. non-cytotoxic. * indicates $p < 0.05$ (from baseline to week 8). CT, chemotherapy; RT, radiotherapy.

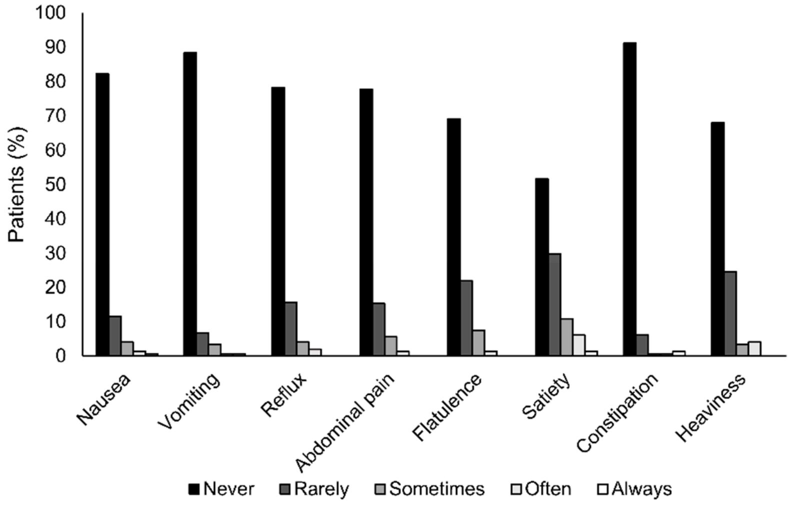

Figure S2. Frequency of tolerability symptoms after 8 weeks of oligomeric enteral nutrition (OEN). The bars show the frequency (never, rarely, sometimes, often, always) of the following tolerability symptoms: nausea, vomiting, reflux, abdominal pain, flatulence, satiety, and constipation.
